# Sepia ink oligopeptide induces apoptosis and growth inhibition in human lung cancer cells

**DOI:** 10.18632/oncotarget.15539

**Published:** 2017-02-20

**Authors:** Zhi Zhang, Lei Sun, Guoren Zhou, Peng Xie, Jinjun Ye

**Affiliations:** ^1^ Department of Thoracic Surgery, Jiangsu Cancer Hospital, Jiangsu Institute of Cancer Research, Nanjing Medical University Affiliated Cancer Hospital, Nanjing 210000, Jiangsu, China; ^2^ Department of Medical Iconography, Jiangsu Cancer Hospital, Jiangsu Institute of Cancer Research, Nanjing Medical University Affiliated Cancer Hospital, Nanjing 210000, Jiangsu, China; ^3^ Department of Chemotherapy, Jiangsu Cancer Hospital, Jiangsu Institute of Cancer Research, Nanjing Medical University Affiliated Cancer Hospital, Nanjing 210000, Jiangsu, China; ^4^ Department of Radiotherapy, Jiangsu Cancer Hospital, Jiangsu Institute of Cancer Research, Nanjing Medical University Affiliated Cancer Hospital, Nanjing 210000, Jiangsu, China

**Keywords:** sepia ink oligopeptide (SIO), lung cancer cell, stable peptide, apoptosis, p53

## Abstract

Sepia ink oligopeptide (SIO), as a tripeptide extracted from Sepia ink, could be used as an inducer of apoptosis in human prostate cancer cells. We designed a cyclo-mimetic peptide of SIO by introducing a disulfide bond to stabilize the native peptide into beta turn structure, and produced a peptide with higher cell permeability and stability. Through labeling an FITC to the N-terminus of the peptide, the cell permeability was examined. Stabilized peptide showed enhanced cellular uptake than linear tripeptide as indicated by flow cytometry and cell fluorescent imaging. The high intracellular delivery of stable SIO could more efficiently inhibit cell proliferation and induce apoptosis. Furthermore, the expression of the anti-apoptotic protein Bcl-2 was down-regulated, whereas pro-apoptotic proteins P53 and caspase-3 were up-regulated by stable SIO. In conclusion, our study is the first to use stable SIO to induce apoptosis in two lung cancer cells A549 and H1299.

## INTRODUCTION

To date, the conventional methods for cancer therapy include surgery, chemotherapy and radiotherapy. Small molecule anti-cancer drugs are the most commonly used chemotherapeutics, which exhibit promising anti-cancer effects [1, 2]. However, the small molecule anti-cancer drugs not only inhibit the growth of cancer cells, but also kill normal cells *in vivo*, especially inducing abnormal function of hematopoietic cells and damaging gastrointestinal mucosal cells. Furthermore, the specificity of small molecule anti-cancer drugs is limited, which can cause off-target and other side-effects. With the accumulation of a large amount of small molecule anti-cancer drugs in the body, the toxicity will be more and more severe, which will likely affect the treatment efficacy and lead to cancer recurrence. Therefore, great research efforts have been devoted to reduce the side-effect of chemotherapy and develop novel anti-cancer drugs with lower toxicity and higher specificity.

Traditional Chinese medicine has been used in China for thousands of years, with good and widely approved clinical efficacy, such as reishi mushrooms [3], American ginseng [4], Astragalus [5], saffron crocus [6] and fel ursi [7] etc. In the last decade, scientists have devoted great effort and indicated that traditional Chinese medicine have potential to inhibit the tumor growth and angiogenesis, which can limit tumor proliferation and block the pathways of tumor migration [8, 9]. With the continuous development of biomedical technology, many active polypeptide substances have been isolated from traditional Chinese medicine. These peptides can not only maintain high efficiency of drug interactions and low toxicity as proteins, but also have lower cost and facile preparation, which has an excellent prospect and opens a new era for treatments of cancer and other diseases [10, 11].

The importance of discovering new peptides has become increasingly recognized in basic studies related to molecular interactions of proteins, as well as clinical investigations of many diseases. Active peptide fraction extracted from Chinese medicine has become a rather important type of new drug modality. Marine resource, as an important origin of traditional medicine, has attracted more and more attention from both scientific and industrial circles. During the past years, products extracted from marine plants and animals were found to have prominent activities against many human cancers [12–14]. For example, Dolastatin-10 and Dolastatins-15, both of which are peptides extracted from *Dolabella auricularia*, have exhibited *in vitro* tumor-suppressor functions [15, 16]. However, the major hurdle for natural peptide products is their limited bioactivity. Usually, oligopeptides are consisted of several amino acids, and its amide bond is susceptible to enzymatic degradation. On the other hand, without a stable structure, it is difficult for oligopeptide to cross the cell membrane. Cyclopeptide is a kind of stabilized peptide, which goes through a feasible cyclization reaction to constrain a peptide into a relative stable conformation, has been proposed to solve the problems of linear natural peptide [17]. The cyclization decreases the conformation variation, while on the other hand increases the anti-proteolytic and anti-metabolization activities.

*Sepia* ink oligopeptide (SIO) is a tripeptide extracted from *Sepia esculenta*, and was reported to exhibit tumor-suppressing function in mouse model of Meth-A fibrosarcoma [18]. It was previously reported that SIO dose-dependently inhibited the proliferation, as well as induced cell death, in prostate cancer cells *in vitro* [19]. In this context, SIO may also possesses antitumor activity in clinical settings, which may serve as an inexpensive therapeutical alternative in the clinical treatment of cancer. In our current study, we investigated the activity of SIO, as well as the underlying mechanisms against human lung cancer, which accounts for the most cancer related deaths worldwide.

## RESULTS

### Synthesis of CSIO peptide

**Figure 1:**
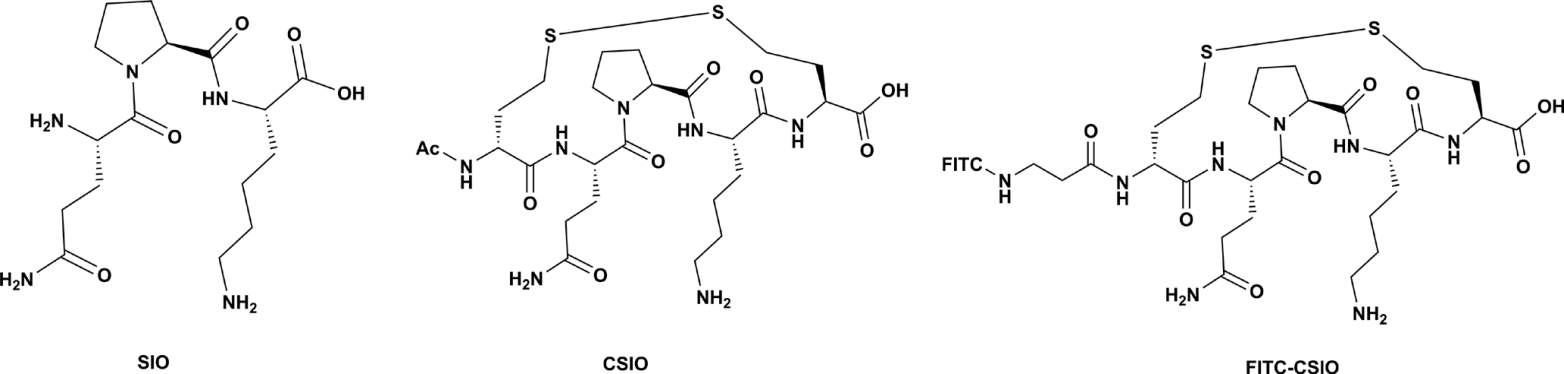
Structures of SIO, CSIO peptide and FITC-Labeled CSIO peptide SIO is consist by three amino acids, CSIO is a mimetic of SIO, which has two extra homocysteine in both terminal of the peptide. FITC-CSIO was a peptide that FITC inked to the N-terminal of peptide via a beta Alanine.

As early as 1982, it was reported that Sepia ink could regulate gastric juice secretion and had anti-ulceration activity [20]. Researchers in Japan found that the peptidoglycan extracted from Sepia ink had higher antitumor activity than the other fractions. In addition, they also found that the carbohydrate part of the peptidoglycan possessed the anticancer activity [21, 22]. Thus Sepia ink peptide has potential in clinical application to treat different diseases. The limited half-life time of peptide in biophysical conditions is the major hurdle for its clinical use, therefore improvement in the bioavailability of Sepia ink peptide is needed. During the past decades, peptide cyclization has been widely used as a strategy to constrain a peptide into fixed conformation, and disulfide bond is the most accessible cyclization method. The disulfide bond is formed by two homocysteine in the oxidation condition. Here, we applied this chemistry to stabilize Sepia ink peptide. In order to keep the active section of peptide, we did not change the origin sequence QPK. Instead, we added two homocysteine to the both termini of QPK. First, we used the SPPS to synthesize the pentapeptide, then an oxidation reaction was performed. HPLC and LC-MS were used to characterize the peptide. For peptide imaging, an FITC was linked to the N-terminus of peptide through a beta-Ala spacer (Figure 1).

### Structure and stability characterization of CSIO peptide

**Figure 2:**
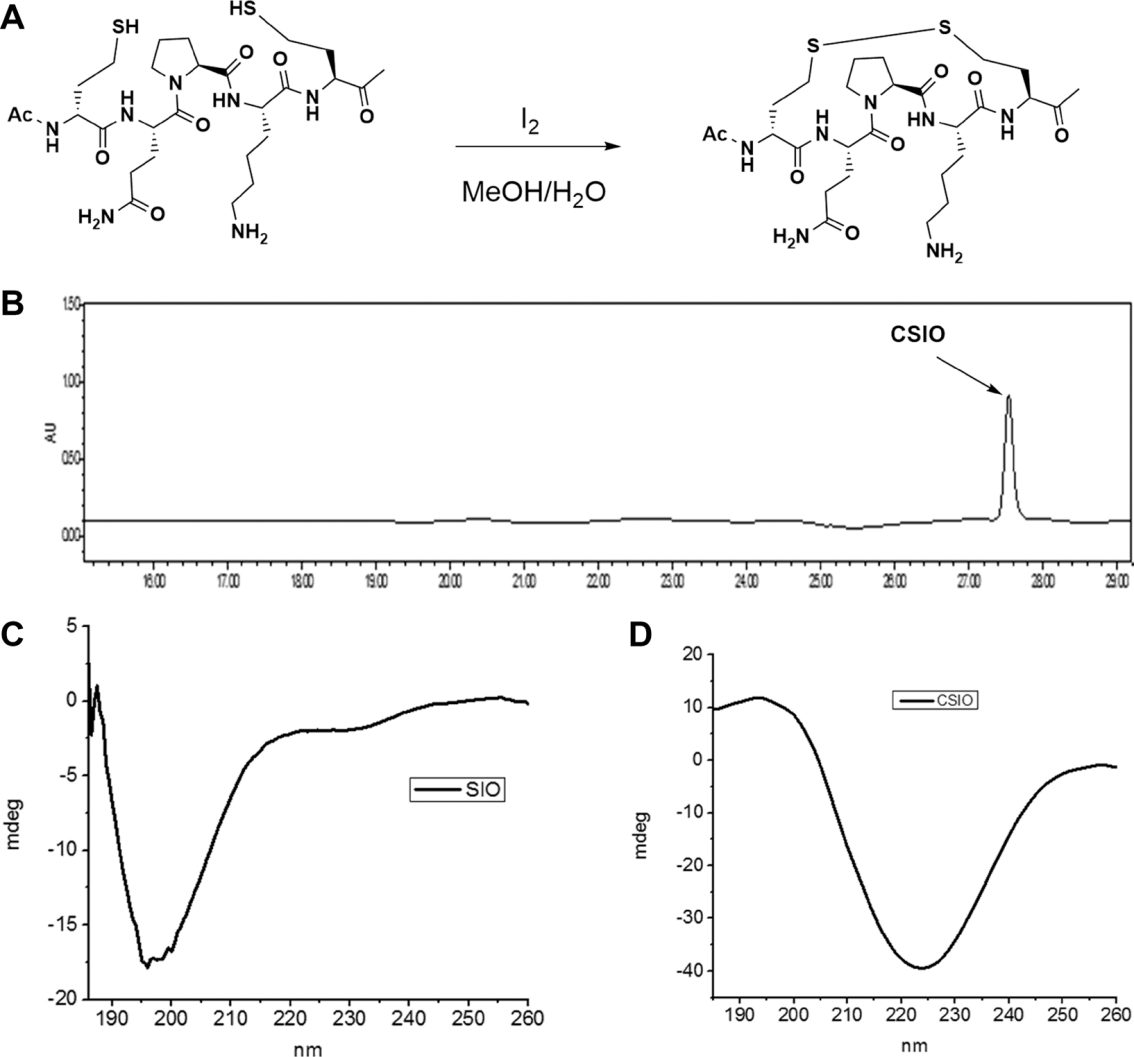
Synthesis and characterization of CSIO peptide (**A**) Schematic shows the oxidation reaction of two homocysteine to disulfide bond. (**B**) The HPLC spectra of CSIO peptide. (**C**, **D**) CD spectra of SIO and CSIO peptides.

**Figure 3:**
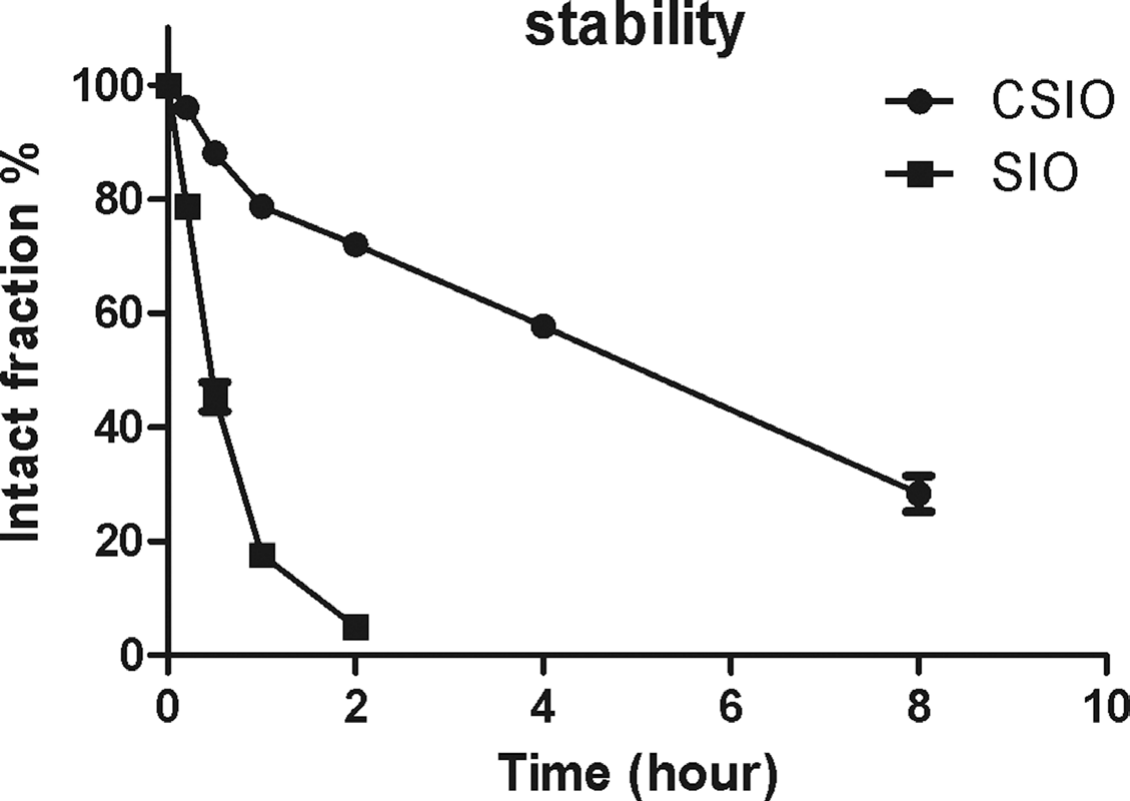
The stability results of CSIO and SIO peptide in cell culture medium Intact fraction of CSIO and SIO were measured at indicated time points and plotted as % of the initial concentration.

A schematic image was presented to show the oxidation reaction of two homocysteine to disulfide bond (Figure 2A), and HPLC spectra of CSIO peptide was shown in Figure 2B. In order to evaluate the conformation change after introducing disulfide bond, CD was used to measure the secondary structure of CSIO and SIO peptides. From the CD spectra, significant structure change was observed. The tripeptide QPK in PBS solution displayed random coil (Figure 2C). While for the CSIO peptide, it presented a turn like structure under the same condition (Figure 2D). In the CD spectra, a maximum negative absorption was observed at 225 nm, which is usually designated to turn or sheet structures. Then the anti-proteolytic property was measured. FITC-labeled peptide was used to detect the stability in serum. The peptide was incubated in human serum for 24 h at 37°C, and aliquot fractions were taken out and monitored by HPLC to check the intact peptide. The results were shown in Figure 3. For SIO peptide, it was quickly degraded in 2 hours, while the t_1/2_ for CSIO peptide is 6 hours, > 10 folds longer than the SIO peptide.

### *In vitro* cellular uptake of linear SIO or stable SIO in lung cancer cells

**Figure 4:**
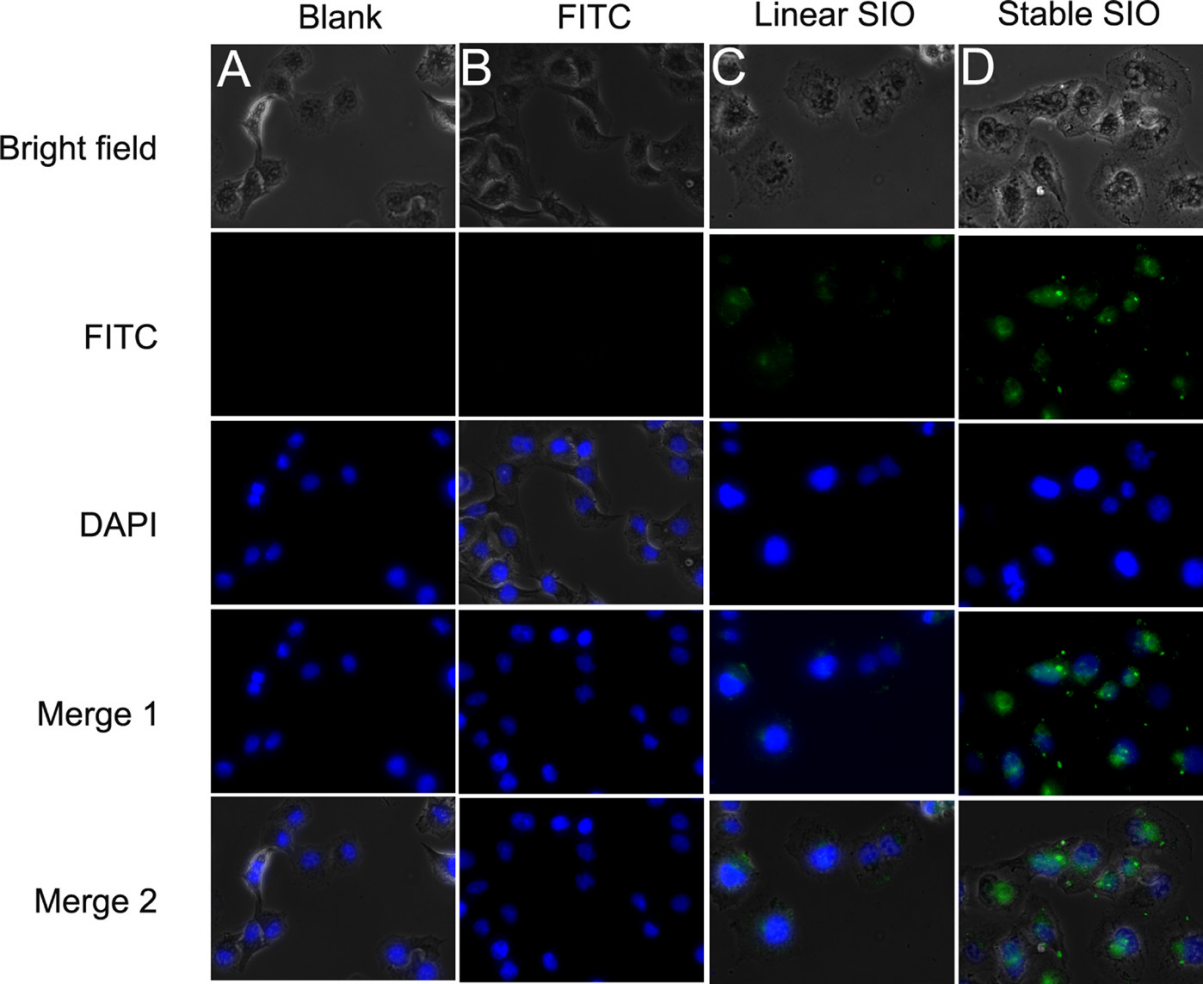
Fluorescent images of A549 human lung cancer cells A549 were treated with PBS (as blank), FITC (5 μM), linear SIO (5 μM) and stable SIO (5 μM) for 2 hours. Peptides are modified with FITC and the signals from peptide-FITC in cells are assigned in green. DAPI is used to label nuclear DNA and are assigned in blue.

Going across the biological membrane is a key factor in biological activity for drug compounds. For many cellular targets, conventional drugs and macromolecular drugs have little effect because of their limited permeability. For peptide drugs, we can easily modulate their permeability through sequence variations. Peptide with positive charges can attach to the cell membrane and undergo endocytosis by the cell membrane. Sepia ink peptide has anticancer effect through inducing cell apoptosis and cell cycle arrest, thus we speculate that enhancing the cellular uptake of SIO could increase its bioactivity. Confocal microscopy was used to evaluate the cell permeability of CSIO peptide. Human lung cancer cell line A549 was treated with PBS (as blank), FITC, stable SIO and linear SIO for 2 hours and then were imaged. The results were shown in Figure 4. Only little fluorescence was detected in cells after treatment with SIO peptide, whereas for CSIO peptide, significant cellular fluorescence was observed, especially in the nucleus. These results indicated that disulfide cyclization increased the cellular uptake of SIO peptide.

### Delivery efficiency of linear SIO or stable SIO into lung cancer cells evaluated by flow cytometry

**Figure 5:**
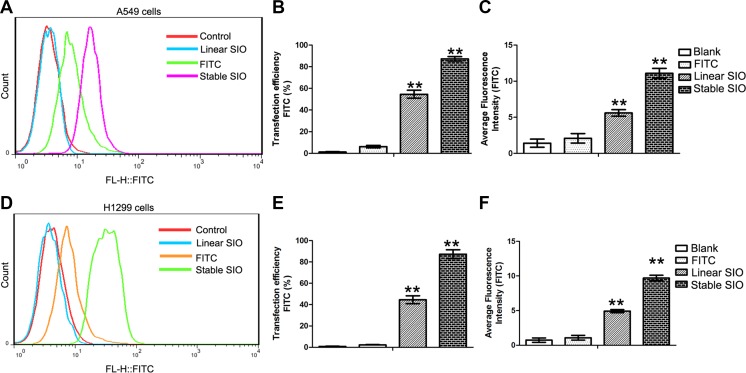
Delivery efficiency of stable SIO or linear SIO in A549 or H1299 cells evaluated by flow cytometry (**A** and **D**) A549 or H1299 cells were treated with PBS (as blank), FITC (5 μM), linear SIO (5 μM) and stable SIO (5 μM) for 2 hours and the statistical count of cells were gathered by flow cytometry. (**B** and **E**) The delivery efficiency and (**C** and **F**) average fluorescent intensity of each group respectively. Average fluorescence intensity presents the FITC intensity in the cells. The delivery efficiency is defined as the ratio between fluorescent cell counts to total cell counts. Data are presented as the means ± SEM of triplicate experiments, **P < 0.01 vs control.

To quantitatively evaluate the corresponding cellular uptakes and average fluorescent intensity of linear SIO or stable SIO in human lung cancer cells, A549 cells were treated with PBS (as blank), FITC, stable SIO and linear SIO for 2 hours and then were analyzed by flow cytometry (Figure 5). Figure 5A shows the statistical count of the FITC intensity in A549 cells after different treatments. Consistent with the fluorescence microscopy results, negligible cellular uptake was observed in cases of FITC (5.82 ± 0.95%) and PBS (as blank, 1.19 ± 0.58%) treated cell groups. Compared to PBS or FITC treated groups, the cells treated with stable SIO exhibited stronger permeability efficiency (91.8 ± 1.87%). However, permeability efficiency from cells treated with linear SIO (58.4 ± 2.91%) were much weaker than that of cells treated with stable SIO, indicating stable SIO possessed the most efficient penetration into cells (Figure 5B). Furthermore, the intensity of the fluorescent signals was consistent with the results of delivery efficiency, which showed that cells treated with stable SIO exhibited the strongest signals than the signals from other groups (Figure 5C). Almost identical results were obtained from another lung cancer cell line H1299 (Figure 5D–5F). Therefore, the results were consistent with the cell fluorescent imaging analysis, which indicated stable SIO was much more appropriate to penetrate into cells.

### Stable SIO can more efficiently inhibit proliferation and induce apoptosis in lung cancer cells

**Figure 6:**
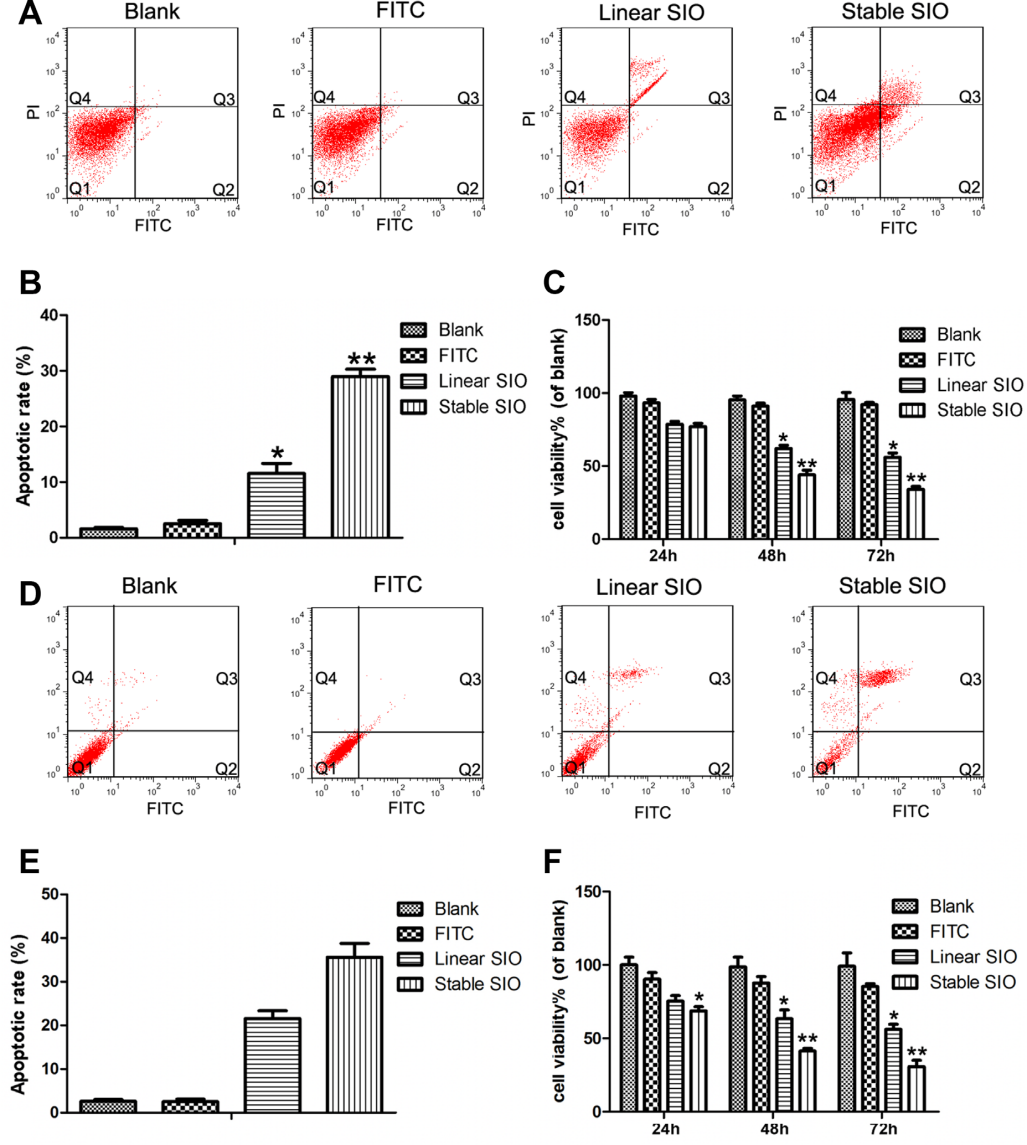
The strong cell penetration of stable SIO in A549 or H1299 cells inhibited cell proliferation and induced cell apoptosis FITC-Annexin V and propidium iodide (PI) containing apoptosis assay on A549 or H1299 cells treated with PBS (as blank), FITC (5 μM), linear SIO (5 μM) and stable SIO (5 μM) for 72 hours. (**A** and **D**) Apoptotic cells stained with Annexin V/PI were measured by flow cytometry. (**B** and **E**) The statistic results of cell apoptosis by flow cytometry. (**C** and **F**) Cell viability of A549 (C) or H1299 cells (F) treated with PBS, FITC, linear SIO and stable SIO for different time (*n* = 3). Percentage cell viability of the treated cells is calculated relative to that of PBS treated cells (with arbitrarily assigned 100% viability). Data are presented as the means ± SEM of triplicate experiments, *P < 0.05, **P < 0.01 vs blank.

Cell proliferation and apoptosis are crucial markers of cancer metastasis and invasive properties. To explore whether the stable SIO has much better effect on inhibiting the proliferation of A549 and H1299 cells, MTT and CCK-8 assays (Supplementary Figure 1) were conducted to investigate the anti-proliferative effect in PBS, FITC, stable SIO and linear SIO treated groups. As shown in Figure 6C, 6F and Supplementary Figure 1, PBS or FITC treated groups showed no obvious inhibition on A549 and H1299 cell growth. On the other hand, the strongest inhibition was observed from the cells treated by stable SIO. It was worth noting that the anti-proliferative effect of SIO was in a time-dependent manner, which was significant at 48 hours (54%), and was peaked at 72 hours (70%).

To further explore the mechanism and examine whether stable SIO could inhibit the proliferation of A549 and H1299 cells by inducing apoptosis, the apoptosis status of A549 and H1299 cells treated with PBS, FITC, stable SIO or linear SIO was assessed by FITC labelled Annexin V and PI staining (Figure 6A and 6B for A549, Figure 6D and 6E for H1299). Cells in Q2 and Q3 zones showed the apoptotic signals, which were stained by Annexin V or Annexin V/PI. Cells stained in Q2 zone with Annexin V indicated the propensity of cells to lose asymmetrical membrane shape in early apoptotic processes. Cells stained in Q3 zone with Annexin V/PI were in late apoptotic processes. Figure 6B and Figure 6E showed the quantitative measurements of the apoptotic cells in the Q2 and Q3 zones of the cells, which suggested the strongest influence on the cell apoptosis by stable SIO. The result indicated that apoptosis induced by stable SIO played an essential role in regulating cell growth and proliferation.

### The expression of the anti-apoptotic protein Bcl-2 and the apoptotic proteins P53 and caspase-3 are regulated by stable SIO

**Figure 7:**
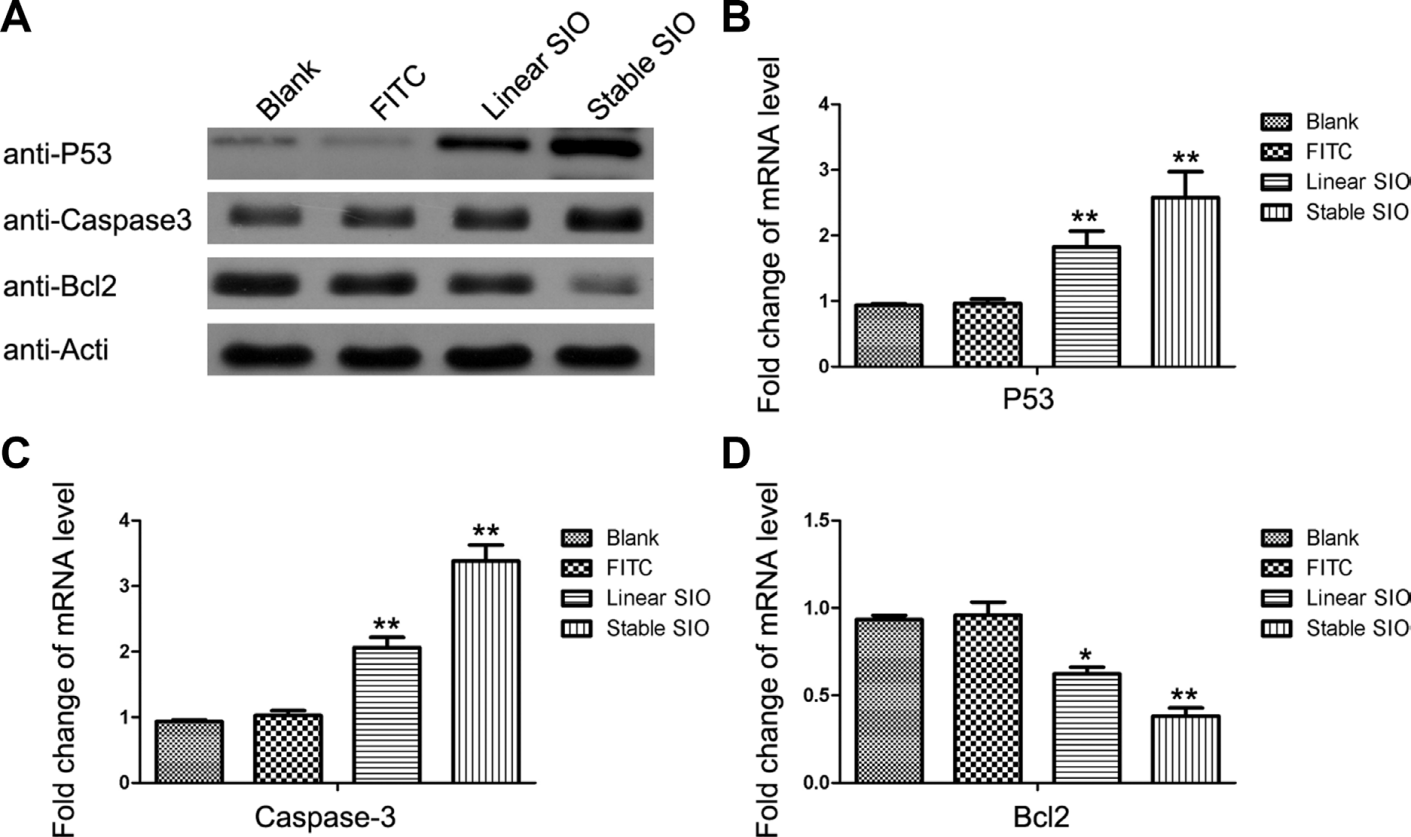
Stable SIO regulated the expression of apoptosis-related genes at mRNA and protein levels A549 cells were treated with PBS (as blank), FITC (5 μM), linear SIO (5 μM) and stable SIO (5 μM) for 72 hours and the mRNA levels and protein levels of apoptosis-related genes P53, Caspase-3 and Bcl-2 were analyzed by Western blot (A) and RT-PCR (**B**–**D**). Data were presented by the mean ± SEM. *P < 0.05, **P < 0.01 vs blank.

After the induction of apoptosis by stable SIO, we further explored whether the expression of apoptosis-related genes were regulated by the stable SIO in lung cancer cells. Therefore, A549 and H1299 cells, respectively, were treated with PBS (as blank), FITC, linear peptide and stable peptide for 2 days. Subsequently, both protein levels and mRNA levels of 3 apoptosis-related genes were analyzed by Western blot and RT-PCR (Figure 7). As expected, anti-apoptotic Bcl-2 expression was down-regulated, and the expressions of the apoptotic genes P53 and caspase-3 were up-regulated by stable SIO. Compared to PBS or FITC treated groups, although linear SIO could influence the expressions of Bcl2, P53 and caspase-3, the effects were weaker than that of stable peptides. In addition to A549 cells, this effect of SIO on the expressions of above proteins could also be reproduced in the other lung cancer cell line H1299 (Supplementary Figure 2). Therefore, stable SIO could more efficiently enter the cells to inhibit cell proliferation by inducing apoptosis.

## DISCUSSION

Except the small molecular drug, there are other anti-cancer drug modalities, including protein drug such as antibody, peptide, nano-medicine and gene medicine. Among them, peptide drugs have many advantages [23]. Due to the intrinsic pharmacological properties, as well as the specificity and safety of peptides, they have promising potential to be used in new clinical therapies. In particular, the efficacy and tolerability of peptide drugs are the major factor in distinguishing peptides from other small molecule drugs. In addition, production of peptide therapeutics is much cheaper than protein-based drugs, owing to the low complexity in peptide structure. Taken together, peptides are the ideal therapeutic alternative that fits in the gap between small molecule drugs and biopharmaceuticals [24].

However there is weakness in peptide drugs as well. First of all, absorption of peptide drugs is poor for oral administration as peptides are difficult to be absorbed in the digestive system [25], therefore injection is the preferred mode of clinical administration. Second, after absorption, the peptides can be rapidly metabolized by proteolytic enzymes, adversely affecting its long-term effects. Third, membrane permeability of peptides is usually not as good as small molecule drugs, making it difficult for the peptides to reach their intracellular targets. Despite the above limitations, peptides do possess the following advantages. First, different from synthetic small molecule drugs, peptides are usually less toxic to cells and organs. Second, they do not accumulate in human body due to the rapid proteolytic degradation. The degradation products are simple amino acids without any cellular toxicity. Peptides can reach their cellular targets with high selectivity, as interactions with the targets are very specific compared to small molecule drugs.

Compared with regular peptides, cyclic peptides exhibit even better advantages [17]. For instance, cyclic peptides exhibit higher biological activity than linear peptides because of their conformational rigidity, which decreases the entropy of the cyclic peptides, enhancing selective binding with their cellular targets such as receptors. Furthermore, cyclic structure, intrinsically without amino or carboxyl terminus, is more resistant to hydrolysis by exopeptidases, and makes it relatively easier to pass plasma membrane of cells as well. Taken together, the above characteristics have made cyclic peptides potentially promising therapeutic agents, and studies are underway to further understand their biological activity to facilitate the synthesis of even better cyclic peptide drugs [1].

Traditional Chinese medicine has been considered as the “naturally combinatorial chemistry libraries”, which are composed of numerous components and are able to act on multiple targets in the human body. The importance of discovering new peptides has become increasingly recognized in basic studies related to molecular interactions of proteins, as well as clinical investigations of many diseases. Increasing the efficacy of these natural products is urgently needed. To date, few reports have been published on improving natural active peptide activity. To the best of our knowledge, we hereby for the first time reported a cyclic SIO peptide [20] mimetic which could significantly improve the stability and bioactivity of the SIO peptide.

In our studies, we have stabilized SIO by disulfide bond strategy and compared the anti-cancer efficiency between stable SIO and linear SIO in A549 and H1299 human lung cancer cells. This work is the first research of stable peptides in traditional Chinese medicines for cancer therapy. With the cyclization modification, stable SIO are sufficiently transported into A549 and H1299 cells, which have been observed by both fluorescent microscope and flow cytometry. The successful penetration into cells has enabled stable SIO to exhibit stronger anti-proliferative effect than linear SIO. To further explore the mechanism, apoptosis assay was conducted, and our results demonstrate that apoptosis is the main cause of the inhibition in cell proliferation by stable SIO. According to the Western blot and RT-PCR assays, the expression of anti-apoptotic gene Bcl-2 has been down-regulated, and the expressions of the apoptotic genes P53 and caspase-3 have been up-regulated by stable SIO. Based on these results, stable peptide engineering could be a promising strategy for constructing potent anti-tumor compounds. While in this article, we only tested the CSIO peptide in two cell lines, more assays are also needed to validate the cancer treatment potency of the CSIO compound.

## MATERIALS AND METHODS

### Chemicals and reagents

The amino acid derivatives used in the current study were purchased from Sigma-Aldrich (St. Louis, MO, USA). Reagents including acetonitrile, methanol, ethanol, diethyl-ether, pyridine, dichloromethane, and DMF (N, N-dimethylformamide) were obtained from Yongda (Tianjin, China).

### Peptide synthesis

100 mg rink amide resin was loaded into a 10 mL peptide synthesis flask, which was then washed sequentially with CH_2_Cl_2_ (3 × 1 ml) and DMF (3 × 1 ml). 2 ml 20% piperidine in DMF was then added to the above reaction flask, which was agitated for 30 min and drained. The resin was then washed sequentially with DMF, CH_2_Cl_2_, MeOH, CH_2_Cl_2_, and DMF (3 × 1 ml each). 1 ml of 4 equiv, 0.15 mmol fmoc-protected amino acids in DMF was added into the reaction flask with 4 equiv, 0.15 mmol HCTU and 8 equiv, 0.3 mmol DIEA, which was agitated for 2 h, followed by sequential washing with DMF, CH_2_Cl_2_, MeOH, CH_2_Cl_2_, and DMF (3 × 1 ml each). The above procedures were repeated until complete coupling of all amino acids to the resin. The oxidation of disulfide bond was performed referred to previous literature. In brief, the synthesized peptide was cleavaged from the resin, and then oxidized with excess iodine in 1: 1 trifluoro- ethanol/water, and the products were isolated by HPLC. For FITC derivative synthesis, a beta alanine was linked to the N-terminus of peptides, then FITC (10 equiv) and DIEA (20 equiv) were mixed with the resin for 12 hours. Finally, the resin was washed with DCM and shrank with MeOH, then used cleavage cocktail (TFA: TIS: H_2_O = 95:2.5:2.5) to cleave the peptide from the resin.

### High performance liquid chromatography

The Shimadzu HPLC system (SHIMAZU Prominence LC-20AT) was used for RP-HPLC analysis. Briefly, crude products were first purified using a Grace Vydac peptide C18 250 × 10 mm column, using gradient elution (0 min 0% acetonitrile, 5 min 20% acetonitrile and 50 min 40% acetonitrile) of 0.1% TFA in water at 1 ml/min flow rate. The purity, stability, aqueous solubility and permeability of the CSIO peptide were measured using analytical LC-MS, with peaks detected at 220 nm.

### Circular dichroism (CD)

SIO and CSIO were dissolved in potassium phosphate solution (pH 7.0) to concentrations of 10–100 μM. The spectra were obtained on an Applied Photophysics Chirascan Circular Dichroism Spectrometer at temperature of 20°C using the following standard measurement parameters: wavelength, 190–250 nm; step resolution, 0.2 nm; speed, 20 nm/sec; accumulations, 10; response, 1 sec; bandwidth, 1 nm; path 3 length, 0.1 cm. Every sample was scanned twice and the final CD spectra was averaged and smoothed using the Pro-data Viewer.

### Stability experiment

The serum solutions were incubated for at 37°C. Aliquots (10 μl) were taken periodically at 5, 10, 30, 45, 60 min, 4 h, 8 h, 24 h, and then 100 μl 12% trichloroacetic acid in H_2_O/CH_3_CN (1:3) was added and cooled to 4°C for 30 min to precipitate serum proteins. The decanted supernatant was analyzed by LC-MS with a 4.6 × 250 mm^2^ Zorbax SB C18 5 μm column, using a 3% per minute linear gradient from 10%–90% acetonitrile over 27 min. The amount of starting material left in each sample was quantified by determination of total ion counts for the molecularion.

### Fluorescence imaging

Stable SIO or linear SIO were first dissolved in DMSO to make a 1 mM stock and then added to cells to a final concentration of 5 μM. The cells were incubated with peptides for 1 hour at 37°C. After incubation, cells were washed 3 times with PBS and then fixed with 4% formaldehyde (Alfa Aesar, MA, USA) in PBS for 10 minutes. They were then washed 3 times with PBS and stained with 1 μg/ml 4′, 6-diamidino-2-phenylindole (DAPI) (Invitrogen, CA, USA) in PBS for 5 minutes. Images of peptide localization in cells were taken on PerkinElmer confocal microscopy. Image processing was done using Volocity software package (Zeiss Imaging, Germany).

### Cell culture

The human lung cancer cell lines A549 and H1299 (American Type Culture Collection) were cultured in F-12K medium (Gibco), supplied with 10% fetal bovine serum, 0.25 μg/ml amphotericin B, 2.4 mM L-alanyl-L-glutamine, 100 μg/ml streptomycin and 100 μg/ml penicillin, at 37°C in a humidified atmosphere with 5% CO_2_.

### Flow cytometry

To test the delivery efficiency of stable SIO or linear SIO, A549 cells were cultured in the 6-well plates and were treated with PBS (as blank), FITC, stable FITC-SIO and linear FITC-SIO separately for 4 hours. After 4 hours, the cells were washed two times with PBS and collected after trypsinization. Cells were then centrifugated at 1,000 rpm for 5 min to remove the trypsin. Samples were then analyzed by the FACSCalibur flow cytometer (Becton Dickinson). The cellular uptake and average fluorescent intensity were analyzed using Flowjo software.

### RNA isolation and quantitative RT-PCR

Total RNA was extracted with TRIzol kit (Invitrogen, Carlsbad, CA, USA), followed by quantitation using Nano Drop 2000. 2 μg of total RNA was used to synthesize cDNA using the reverse transcriptase kit (Takara, Japan), which was then quantified by RT-PCR. The primers were provided in the Table 1.

**Table 1:** RT-PCR primers

Primer name	Orientation	Sequence (5′-3′)
Human P53	Forward	CGGTTTCCGTCTGGGCTTCT
Human P53	Reverse	CAACCTCAGGCGGCTCATAG
Human Caspase-3	Forward	GTGGAATTGATGCGTGATGT
Human Caspase-3	Reverse	TAACCAGGTGCTGTGGAGTA
Human Bcl2	Forward	TGTGGTCCACCTGACCCTCC
Human Bcl2	Reverse	CATCCCAGCCTCCGTTATCC
Human β-Actin	Forward	GTCACCAACTGGGACGACAT
Human β-Actin	Reverse	GCACAGCCTGGATAGCAACG

### Apoptosis analysis

The apoptosis assay was conducted using an Annexin V: FITC Apoptosis Detection Kit I (BD Pharmingen) according to the manufacturer's instructions. Briefly, cells were collected and washed two times with PBS, collected after trypsinization, and resuspended in binding buffer. Subsequently, transferring 100 μl of the solution to a 5 ml culture tube and added 5 μl of FITC Annexin V and 5 μl PI in cells for 15 min at RT. The stained cells were subjected to flow cytometric analysis to measure the apoptotic cells.

### Western blotting

For Western blot analysis, A549 and H1299 cells were treated with PBS (as blank), FITC, stable FITC-SIO and linear FITC-SIO for 72 hours as described for the RT-PCR assay. To isolate protein, cells were washed with PBS and harvested using the lysis buffer (50 mM Tris·Cl pH = 6.8, 2% SDS, 6% Glycerol, 1% β-mercapitalethanol, 0.004% bromophenol blue). The denatured cellular extracts were resolved by 12% SDS-PAGE gels. Protein bands in the gel were then transferred to Nitrocellulose Blotting membranes and incubated with the appropriate primary antibody overnight. The antibodies were purchased from Abcam and used in dilutions as follows: 1:1,000 for P53 (ab32389), 1:1,000 for P21 (ab109520), 1:1,000 for Bcl2 (ab32124) and 1:1,000 for actin (ab8226). Goat anti-rabbit or anti-mouse secondary antibodies were used for secondary incubation for 1 hour at room temperature.

### Statistical analysis

Data are presented as the means ± SEM of triplicate experiments, **P < 0.01 vs control. All statistical calculations were performed with the SPSS 11.0 software package. Two-way ANOVA was used for statistical analysis, and statistical significance is accepted at **p ≤ 0.01 which represents very significant differences.

## SUPPLEMENTARY FIGURES


