# Supplementary material for: Sepia ink oligopeptide induces apoptosis and growth inhibition in human lung cancer cells
Source: Oncotarget. 2017 Feb 20;8(14):23202–12. doi: 10.18632/oncotarget.15539 (PMC5410297; doi:10.18632/oncotarget.15539)
Supplement: Supplementary file 1 [file oncotarget-08-23202-s001.pdf]

Sepia ink oligopeptide induces apoptosis and growth inhibition in human lung cancer cells

Supplementary Materials

SUPPLEMENTARY INFORMATION

MATERIALS AND METHODS

Cell viability was measured using Cell Counting Kit-8 (CCK-8, Sigma, St. Louis, MO, USA) following the procedure described in Sigma. A549 and H1299 cells were seeded at 5 × 10^3 per well into 96-well plates and allowed to adhere for 24 h. Cells were treated with different peptides for 48 and 72 h. 10 μL CCK-8 solution was added and the cells were incubated for 4 h at 37°C with 5% CO2. The plates were processed as described above, and the OD_{450} of the supernatant was measured.

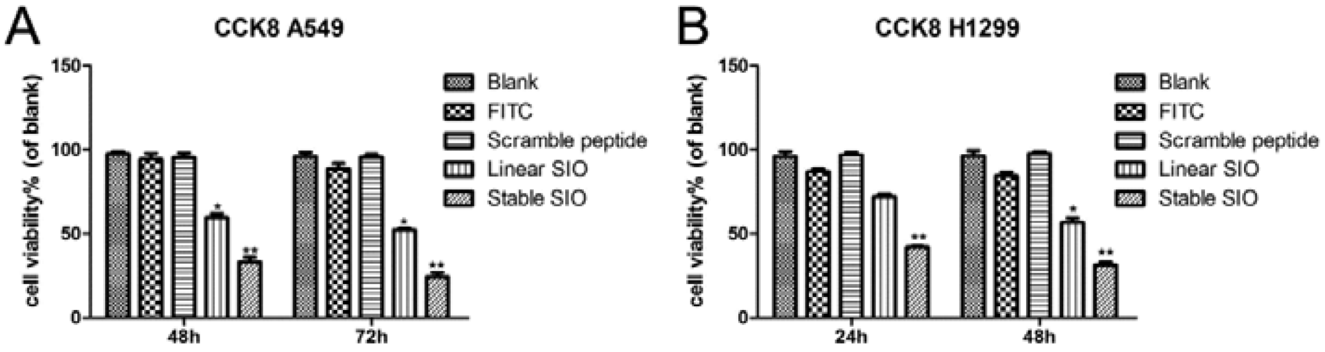

Supplementary Figure 1: Cell viability of A549 (**A**) or H1299 cells (**B**) treated with PBS, FITC, linear SIO and stable SIO for different time (*n* = 3). WST-8 signal (measured at 450 nm) were calculated for the percentage cell viability of the treated cells compared to PBS treated cells (with arbitrarily assigned 100% viability). Data are presented as the means ± SEM of triplicate experiments, $^{*}P < 0.05$, $^{**}P < 0.01$ vs blank.

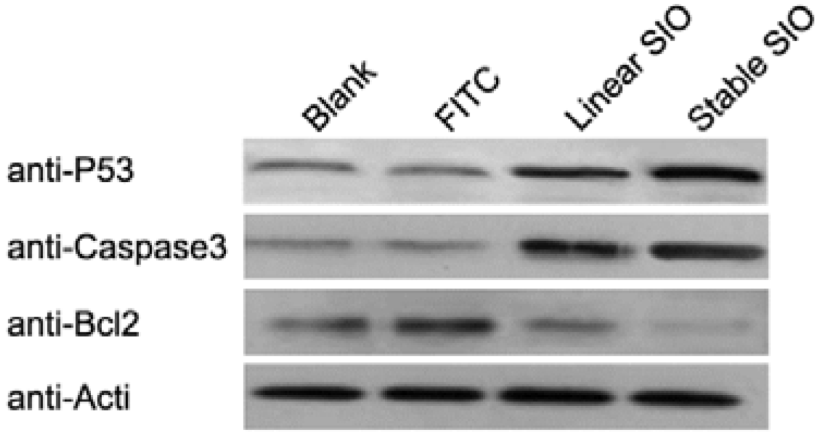

Supplementary Figure 2: H1299 cells were treated with PBS (as blank), FITC (5 µM), linear SIO (5 µM) and stable SIO (5 µM) for 72 hours and the protein levels of apoptosis-related genes P53, Caspase-3 and Bcl-2 were analyzed by Western blot.
